# Primary Ductal Her-2 Positive Adenocarcinoma of Salivary Gland: A Long Follow-Up Case Report and Review of the Literature

**DOI:** 10.1155/2024/4410206

**Published:** 2024-09-12

**Authors:** C. L. Deantoni, M. Midulla, A. Mirabile, A. Chiara, R. Lucchini, L. Giannini, M. Torrisi, A. Fodor, N. G. Di Muzio, I. Dell'Oca

**Affiliations:** ^1^ Radiation Oncology Department IRCCS San Raffaele Scientific Institute, Milan, Italy; ^2^ Department of Otorhinolaryngology IRCCS San Raffaele Scientific Institute, Milan, Italy; ^3^ Vita-Salute San Raffaele University, Milan, Italy

## Abstract

**Background:** Epithelial tumors of lacrimal glands are rare and primary ductal adenocarcinoma of the lacrimal gland accounts for only 2% of all epithelial lacrimal gland tumors. Considering its rarity and lack of uniform diagnostic criteria, treatment protocols are not well defined.

In this study, we describe a Her-2 positive case and review previously reported cases.

**Methods:** In 2012, a 42-year-old woman affected by primary ductal adenocarcinoma of the lacrimal gland was treated with transpalpebral anterior orbitotomy and adjuvant radiotherapy. In July 2013, she presented local relapse and she underwent orbital exenteration. In November 2013, for neck nodal progression, seven cycles of chemotherapy (cisplatin and epirubicin) associated with a humanized monoclonal antibody–targeting HER 2 therapy (trastuzumab and pertuzumab) were performed, with a marked response rate. Then, she underwent total parotidectomy with right neck lymphadenectomy and adjuvant hadrontherapy.

**Results:** Nine years later (113 months) after treatment completion, the patient was alive without disease and with acceptable toxicity.

**Conclusions:** In primary ductal adenocarcinoma of the lacrimal gland, early diagnosis and multimodal treatments could be crucial, considering its often aggressive tendency. Considering the lack of treatment guidelines, case report recording can be useful in patient management.

## 1. Introduction

Epithelial tumors of lacrimal glands are rare, and they occur in about 1 in 1,000,000 individuals per year and constitute 5%–25% of all orbital malignancies [1–3]. More than 30 different subtypes were described in the 2005 WHO Classification of Tumors [4]. Primary ductal adenocarcinoma of the lacrimal gland (lgPDA) accounts for only 2% of all epithelial lacrimal gland tumors [3]. Due to their typical highly aggressive behavior, the diagnosis in early stage and long-term follow-up are required. Considering its rarity and lack of uniform diagnostic criteria, treatment protocols are not well defined. According to the literature [5], gross cystic disease fluid protein 15 (GCDFP-15), androgen receptor (AR), and human epidermal growth factor receptor 2 (Her-2) should be tested as biomarkers to confirm the diagnosis, guide therapy, and further predict prognosis. In this study, we describe a Her-2 positive case and review previously reported cases. Ethics approval was waived by our Institutional Ethics Committee, in view of the retrospective nature of the study. All the procedures described had been performed for routine patient management and care. The study was performed in accordance with the ethical standards of the Helsinki Declaration.

## 2. Case Report

**Figure 1:**
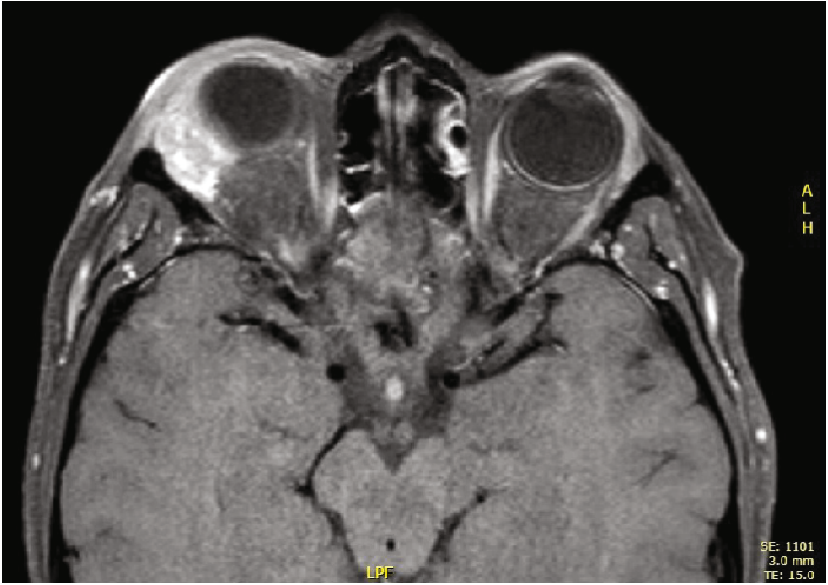
Contrast-enhanced magnetic resonance imaging of the orbits showed a 2 × 1 cm lesion involving the right lacrimal gland with displacement of the lateral rectus muscle and the superior muscles group.

In December 2012, a 42-year-old female presented progressive swelling of the eyelid and exophthalmos. Maxillofacial CT and orbit MRI examinations revealed a 2 × 1 cm lesion involving the right lacrimal gland with tiny specks of calcification in the center and heterogeneous enhancement. The lateral rectus muscle and the superior muscle group were displaced (Figure 1). No pathological nodes were found with the neck ultrasound examination.

On February 14, 2013, the patient underwent a transpalpebral anterior orbitotomy with en block removal of the lesion. Histopathological examination revealed adenocarcinoma with focal positive margins (pT1pNxR1). The tumor cells were immunoreactive for cytokeratin-7 (CK7), p63, AR (1+), and Her-2 (1+). Immunostains for estrogen receptor (ER), progesterone receptor (PgR), cytokeratin-20 (CK20), and thyroid transcription factor-1 (TTF-1) were negative. MIB-1 index was not investigated.

Whole body positron emission tomography (PET)-CT showed no evidence of systemic involvement.

The patient received adjuvant radiotherapy on the tumor bed. In order to spare organs at risk (the retina in particular), a dose of 50 Gy in 25 fractions was delivered, with image-guided–helical intensity-modulated radiotherapy (IG-IMRT) technique. The target volume was defined by the fusion of CT and preoperative MRI. The treatment was performed from April 8, 2013, to May 13, 2013, without interruptions. Conjunctivitis G2 and erythema G2, according to CTCAE v 4.1 [6], were registered during radiotherapy.

**Figure 2:**
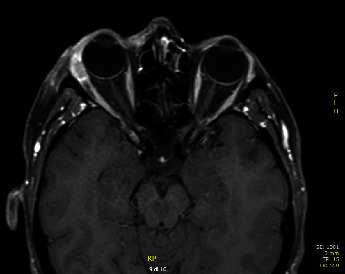
Magnetic resonance imaging showing local relapse in the form of multiple millimetric spread inside the superior orbital muscle.

In July 2013, orbit MRI demonstrated local relapse in the form of multiple millimetric spread inside the superior orbital muscle (Figure 2). The biopsy was positive for adenocarcinoma. In October 2013, PET-CT imaging showed increased metabolic activity of the right orbital soft tissue mass and no evidence of distant metastasis. On October 10, 2013, the patient underwent orbital exenteration. In November 2013, a biopsy of a right retroauricular node was performed and confirmed nodal involvement. An ultrasound examination revealed pathological neck nodes (retroauricular; IIa and IIb levels). From November 2013 to March 2014, the patient underwent seven cycles of chemotherapy with cisplatin and epirubicin [7–9] associated with a humanized monoclonal antibody-targeting HER 2 therapy (trastuzumab and pertuzumab). Subsequent MRI and PET showed a marked response rate to the performed therapy. On April 2, 2014, total parotidectomy and right neck lymphadenectomy were performed: 4/14 nodes were positive. After surgery, adjuvant hadrontherapy was performed. From May 12, 2014, to June 12, 2014, the right orbit and right lateral cervical lymph nodes area were irradiated with carbon ion radiotherapy; the prescribed dose was 60 Gy(RBE) delivered in 20 fractions (four fractions/week) with two beams optimized simultaneously ((IMPT) intensity-modulated particle therapy). Then, from June 16, 2014, to June 24, 2014, right lateral cervical lymph node areas were irradiated with proton radiotherapy performed by single beam optimization; the prescribed dose was 14 Gy (RBE) delivered in seven fractions (five fractions/week).

The patient continued to be on regular periodic follow-ups and was doing well without any further tumor recurrence. Nine years later (113 months) after treatment completion, the patient was alive without disease. She presents an unchanged 5-mm skin fistula located in the middle canthus. Other late toxicities registered were sporadic electric shock-type dysesthesia (neuropathy G1) and xerostomia G1 [6].

## 3. Discussion

lgPDA was first described by Katz et al. in 1996 [10]. To the best of our knowledge, 37 cases have been described, and only 18 HER-2 positive cases have been reported in literature in the past 27 years.

We retrospectively reviewed 18 collected case reports and our case of HER-2 positive lgPDA (Tables 1 and 2). The median age was 59 years (39–77), and most cases were males (70.6%). The median follow-up was 24 months (2–120). The most common presenting symptoms are proptosis, swelling, and exophthalmos. The typical CT appearance of this tumor is an irregular lesion with focal destruction of the lacrimal gland area and, not rarely, invasion of extraocular muscle or bone. In our case and in others described previously [15, 16], calcifications inside the tumor are present.

Early diagnosis is crucial because the tumor appears to be pretty aggressive. Although the tumor usually does not initially present with locoregional or distant metastasis, local recurrence, nodal, or distant metastasis may subsequently develop in 3%, 24%, and 50%, respectively [11]. Pathological examination of lgPDA often shows a human EGFR-2 (HER-2) receptor positivity, while ER and PgR are often not expressed [21]. According to this, we can consider lgPDA more similar to salivary duct carcinomas than to breast carcinoma. Clinically, aggressive presentation of salivary duct and lacrimal gland carcinomas is similar, with about half of patients developing distant metastasis. Therefore, the treatment for lgPDA is borrowed from the treatment for salivary duct carcinomas, and the majority of efficacy data are referred to the latter.

Lacrimal gland carcinomas are associated with poor local control and significant morbidity and mortality rates [22]. To date, no guidelines on standard treatment have been developed and the majority of patients underwent surgery (tumor resection or exenteration) followed by radiotherapy [23, 24]. However, local recurrence, distant relapse, and cancer-related mortality risks are higher after orbital exenteration [23]. Thus, eye-preserving surgery followed by adjuvant RT has recently gained popularity, but the optimal timing and approach remain the subject of debate [22, 23].

Proton therapy or even heavy ion therapy applied to lacrimal gland cancer is considered promising for reducing low doses delivered outside the treatment fields [25] because of both sleeper dose gradients and, in the case of heavy ions, a higher biological effectiveness [26].

The efficacy of chemotherapy was uncertain as there are only few cases receiving adjuvant chemoradiotherapy [11, 27, 28]. Due to their rarity, the effectiveness of chemotherapy in lgPDA requires further investigations.

Additionally, there are some studies that androgen deprivation therapy could be beneficial for patients with recurrent or disseminated salivary gland carcinoma [29]. A case of lgPDA (pT2NxM1) treated by total androgen blockage therapy was reported in the literature: The patient survived more than 10 months [15].

Dennie [14] reported a case of metastatic lgPDA treated with Lapatinib (tyrosine kinase inhibitor against HER-2 and EGFR) and survived 4 years after diagnosis.

The HER2 protein expression or gene amplification is found in about 30% of salivary duct carcinomas and is associated with poor prognosis, suggesting HER2 as a potential therapeutic target. However, a single HER2-targeted agent has shown only modest efficacy in HER2 overexpressing salivary gland carcinoma, prompting interest in combinatorial approaches to overcome the limitation of single-agent approaches [30]. Recently, dual HER2 inhibition with trastuzumab with pertuzumab, an HER2 dimerization inhibitor antibody, demonstrated superior antitumor efficacy in HER2-positive breast cancer in both neoadjuvant and metastatic settings with improved overall survival over single HER2 blockade with trastuzumab. Recent case reports demonstrated a promising response of HER2-positive salivary duct carcinoma to dual HER2 blockade [31]. In the literature, only four patients with lacrimal gland tumors treated with target therapy against HER-2 are reported. Three of them were alive with disease, but with a very short follow-up (3, 10 months, and not registered for follow-up) [11, 12, 20], and the third one [14] died after 48 months from the primary tumor presentation. Our patient, 109 months after all treatment completion, is alive without evidence of disease and refers to a very good quality of life. To the best of our knowledge, this is one of the first cases in which anti-Her-2 antibody was used in this setting of patients and the one that better demonstrated that an aggressive treatment that also comprises these agents may bring to complete response in recurrent lgPDA. Very recently, MyPathway multiple basket study concluded that HER2-targeted therapy may have utility in a variety of KRAS wild-type, HER2-amplified, and overexpressed solid tumors [32, 33].

Even with multimodal and aggressive treatments, at the time of their case report publication, only four patients were collected, and the one we now reported was free from disease.

## 4. Conclusions

lgPDA is a high-grade epithelial tumor similar to salivary ductal carcinomas. Early diagnosis could be crucial, considering its often aggressive tendency and the lack of treatment guidelines. The registration of all cases treated all over the world could be important in order to better learn to treat this rare tumor.

## Figures and Tables

**Table 1:** Clinical features, receptor status, and staging of 17 published cases and our one new case of primary ductal HER-2 positive adenocarcinoma of the lacrimal gland.

**Case**	**First author, year**	**Age**	**Sex**	**Complaint**	**TNM**	**Size (mm)**	**AR**	**ER**	**PgR**
1	See [11], 2019	62	M	Swelling of upper eyelid	T2N0M0	38	+	NA	NA
2	See [11], 2019	61	M	Pain, redness of upper lid	T1aN1M0	19	+	NA	NA
3	See [11], 2019	74	F	Proptosis	T2N0M0	23	+	NA	NA
4	Patel [12], 2018	54	M	Decreasing vision and proptosis	T2N1M1	34	+	NA	NA
5	Andreasen [13], 2017	77	M	Xherophtalmia, diplopia, and blepharitis	T4bN0M0	32	+	—	—
6	Andreasen [13], 2017	53	M	Lumbar pain, severe headache, and proptosis	T1N0M1	20	+	—	—
7	Andreasen [13], 2017	73	M	Progressive inferonasal globe displacement and proptosis	T4bN0M0	30	−	—	—
8	Dennie [14], 2015	53	F	Progressive headache, blurring of vision, and proptosis	T4cN0M0	30	NA	—	—
9	Zhu [5], 2015	49	F	Painless, palpable mass, double vision, and epiphora	T1N0M0	11	+	—	—
10	Ricci [15], 2014	71	M	Lumbar pain and exophthalmos	T2NxM0	23	+	—	—
11	Kubota [16], 2013	67	M	Upper eyelid swelling	T4bN1M0	28	+	—	—
12	Kubota [16], 2013	39	M	Upper eyelid swelling	T4bN0M0	25	+	—	—
13	Kubota [16], 2013	46	F	Upper eyelid swelling	T2N0M0	25	+	—	—
14	Damasceno [17], 2012	78	M	Diplopia, painless, palpable mass, and restricted adduction	T2N0M0	24	NA	NA	NA
15	Takahira [18], 2007	48	F	Progressive exophthalmos	T2N0M0	38	+	—	—
16	Tripathy [19], 2021	66	M	Swelling and proptosis	T2aN0M0	29	+	—	—
17	Tripathy [19], 2021	58	M	Swelling and proptosis	T3N1M0	21	+	—	—
18	Mansi [20], 2023	60	M	Back pain and proptosis	M1	NA	NA	—	NA
19	Our case	42	F	Swelling and exophthalmos	T1NxM0	20	+	—	—

Abbreviations: AR = androgen receptor; ER = estrogen receptor; PgR = progesterone receptor.

**Table 2:** Treatment and oncological status of 17 published cases and our one new case of primary ductal HER-2 positive adenocarcinoma of the lacrimal gland.

**Case**	**First author, year**	**Surgery**	**Radiotherapy**	**Chemotherapy**	**Immunotherapy**	**Metastasis**	**Follow-up**	**Status**
1	See [11], 2019	TR	No	Yes	Trastuzumab	/	3	AWD
2	See [11], 2019	ET + nodal biopsy	No	No	No	Neck nodes	2	AWD
3	See [11], 2019	TR	Yes	No	No	/	12	AOD
4	Patel [12], 2018	TR	No	Yes	Trastuzumab	Ipsilateral parotid gland and liver	10	AWD
5	Andreasen [13], 2017	ET	Yes	No		/	19	DOC
6	Andreasen [13], 2017	no	No	No	No	Diffuse spread of metastasis	60	DWD
7	Andreasen [13], 2017	TR	Yes	No	No	/	17	DWD
8	Dennie [14], 2015	TR	Yes	No	Lapatinib	Vertebrae and cerebellum	48	DWD
9	Zhu [5], 2015	ET	Yes	No	No	/	9	AOD
10	Ricci [15], 2014	ET	Yes	No	Androgen blockade	Lumbar spine	19	AWD
11	Kubota [16], 2013	Node resection	Yes	No	No	Cervical node and lung	24	DWD
12	Kubota [16], 2013	ET	Yes	No	No	Lung and brain	120	AWD
13	Kubota [16], 2013	TR	No	No	No	/	66	AOD
14	Damasceno [17], 2012	ET	No	No	No	Parotid and cervical nodes	24	DWD
15	Takahira [18], 2007	TR	Yes	No	No	/	NA	NA
16	Tripathy [19], 2021	ET	Yes	Yes	No	Brain	72	AWD
17	Tripathy [19], 2021	TR+ node resection	Yes	Yes	No	Bladder and brain	36	DWD
18	Mansi [20], 2023	no	Yes (palliation)	Yes	Trastuzumab	NA	NA	AWD
19	Our case	TR➔ET	Yes (also reirradiation)	Yes	Trastuzuab + pertuzumab	/	113	AOD

Abbreviations: AOD = alive without disease; AWD = alive with disease; DOC = dead of other cause; DWD = lgPDA-related death; ET = exenteration; TR= tumor resection.

## Data Availability

The data that support the findings of this study are available on request from the corresponding author to researchers who provide a methodologically sound proposal. Requests made to the corresponding author (C.L.D.) will be evaluated by the IRCCS San Raffaele Scientific Institute Ethics Committee.
